# Current Measurement Transducer Based on Current-To-Voltage-To-Frequency Converting Ring Oscillator with Cascade Bias Circuit

**DOI:** 10.3390/s20020493

**Published:** 2020-01-15

**Authors:** Jongha Park, Jung-Hyun Park, Seong-Ook Jung

**Affiliations:** Department of Electrical & Electronic Engineering, Yonsei University, Seoul 03722, Korea; ninetail234@yonsei.ac.kr (J.P.); 87pjh@yonsei.ac.kr (J.-H.P.)

**Keywords:** current-to-frequency (I–F) converting current transducer, current-to-voltage-to-frequency (I–V–F) converting current transducer, ring oscillator (RO), biosensor

## Abstract

We propose a ring oscillator (RO) based current-to-voltage-to-frequency (I–V–F) converting current transducer with a cascade bias circuit. The I–V–F converting scheme guarantees highly stable biasing against RO, with a rail-to-rail output operation. This device was fabricated using National NanoFab Center (NNFC) 180 nm complementary metal-oxide-semiconductor (CMOS) technology, which achieves a current resolution of 1 nA in a measurement range up to 200 nA. A noise floor of 11.8 pA/√Hz, maximum differential nonlinearity (DNL) of 0.15 in 1 nA steps, and rail-to-rail output with a 1.8 V power supply is achieved. The proposed transducer can be effectively applied to bio-sensing devices requiring a compact area and low power consumption with a low current output. The fabricated structure can be applied to monolithic-three-dimensional integration with a bio-sensing device.

## 1. Introduction

**Figure 1:**
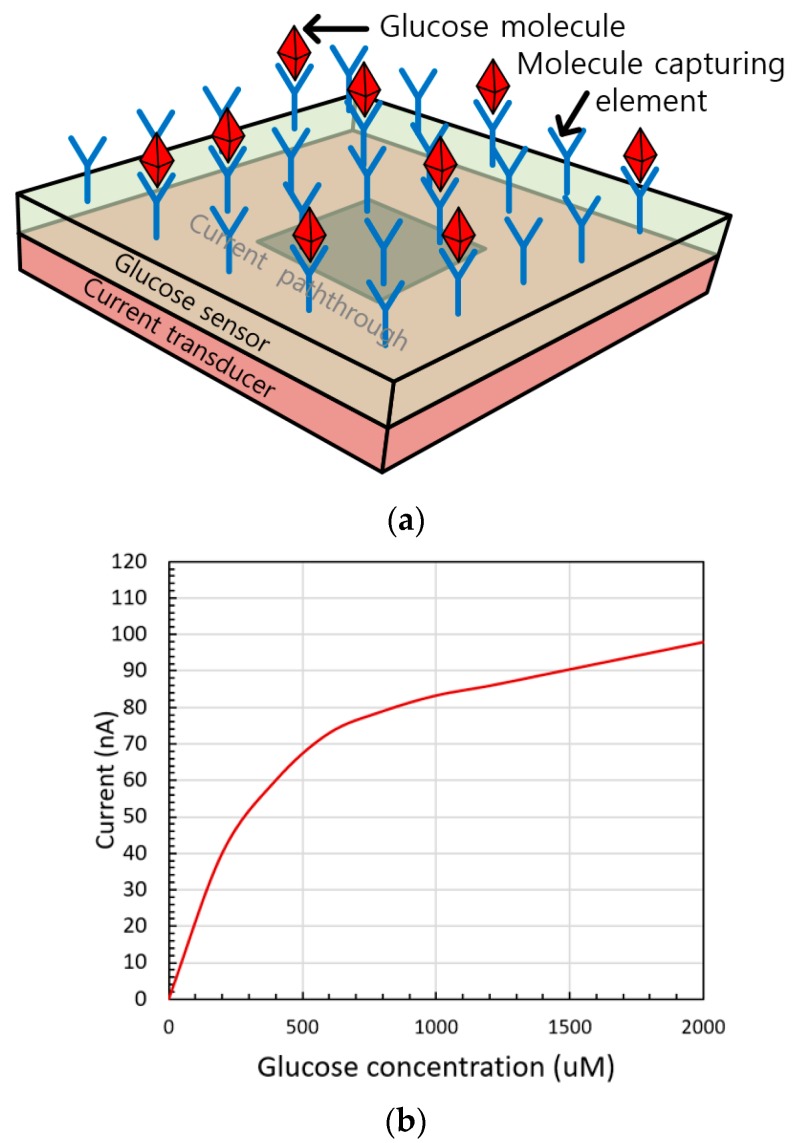
(**a**) Glucose detecting biosensor integrated with a current transducer; (**b**) output current of a glucose sensor according to the glucose concentration.

In various bio-sensing applications, including glucose concentration monitoring and neurotransmitter detection [1], precise current measuring is essential. For example, glucose-detecting biosensors can be designed as amperometric sensors. As the element capturing glucose molecules interacts with the target molecule, it reacts on the electrode surface, producing charged species [2]. The amperometric sensor then generates a current output in the order of tens of nanoamperes, proportional to the glucose concentration, as shown in Figure 1. For precise measurement of the glucose concentration, a current measurement transducer with a resolution of a few nanoamperes should be designed [3]. There are several voltage-controlled oscillator based analog-to-digital converters that convert the amperometric input to the frequency output with great linearity characteristics and resolution [4,5]. However, large area and power consumption relative to the glucose sensor causes overhead to the integrated device as the size of the glucose sensor unit is μm scale [2]. Therefore, the current measurement transducer should be designed with low power, low complexity, and a size suitable for integration with the glucose sensor. A current-starved ring oscillator (RO)-based current-to-frequency (I–F) converter was designed to satisfy these criteria [6], but the direct I–F conversion induces current fluctuation, resulting in linearity error, and the pull-down structure does not guarantee the rail-to-rail output.

This paper introduces an RO-based I–F converting current transducer structure featuring a cascade bias circuit. The proposed transducer exhibits improved current resolution using a current-to-voltage-to-frequency (I–V–F) conversion scheme and achieves a fully swinging output voltage using a direct pull-down structure. The proposed current transducer was fabricated using the National NanoFab Center (NNFC) 180 nm complementary metal-oxide-semiconductor (CMOS) process, where monolithic three-dimensional (M3D) integration is applicable. M3D integration enables heterogeneous integration [7], simplifying the assembly of tiers requiring different fabrication processes. In this vertical integration, the area occupation is determined by the preponderant size of the two circuits, leading to the maximum area compactness.

## 2. Previous I–F Converting Current Transducer

**Figure 2:**
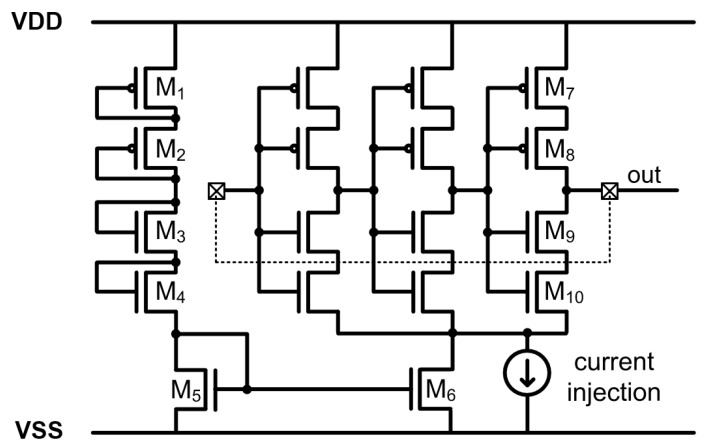
Current starved ring oscillator (RO)-based current-to-frequency (I–F) converting current transducer.

**Figure 3:**
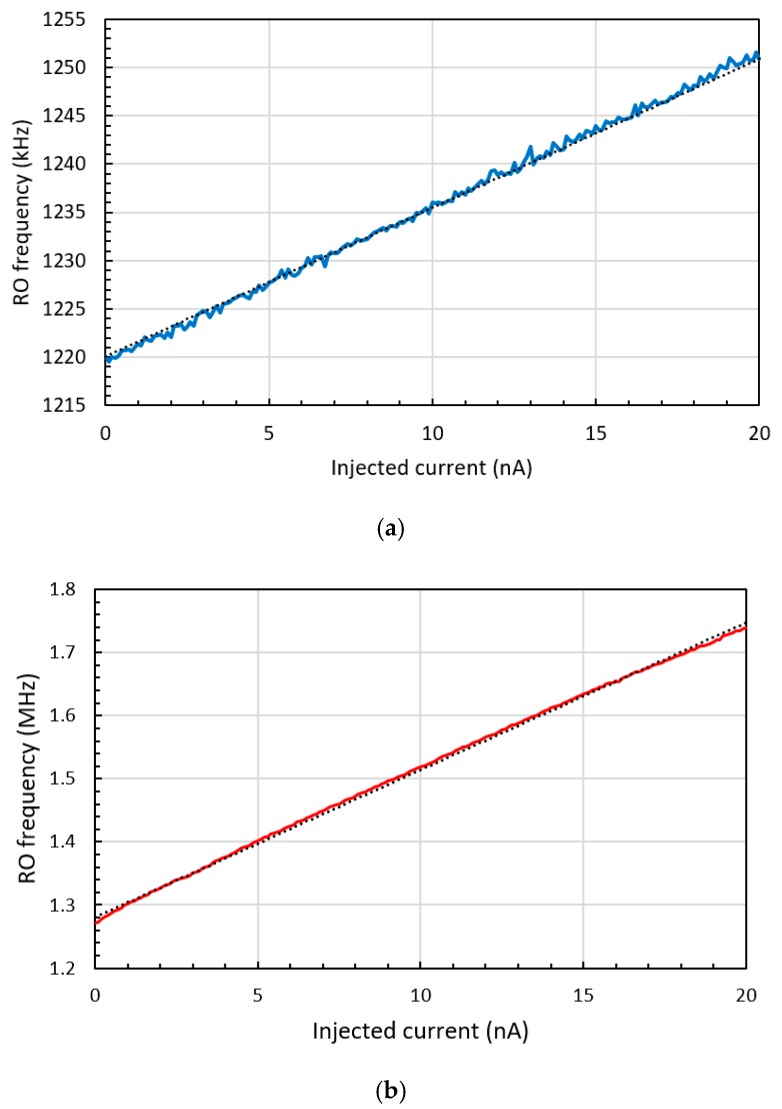
Frequency output of the (**a**) three-stage I–F and (**b**) 21-stage current-to-voltage-to-frequency (I–V–F) converting current transducer.

An I–F converter with a current-starved RO and current mirror bias circuit was previously reported, as shown in Figure 2 [6]. The bias circuit, with a cascade of diode-connected transistors (M_1_–M_4_), was used as a constant current source, and a single-stage current mirror (M_5_ and M_6_) was used for the RO biasing. The current starved RO is composed of stacked inverters (M_7_–M_10_ and their corresponding transistors in different inverters) to produce a long delay and high resistance. As the measurement-required current directly biases the RO, the RO oscillates with a frequency proportional to the sum of the fixed biasing (M_1_–M_6_) and injected currents, as shown in Figure 3a. By observing the RO frequency, the injected current can be measured by the inverse calculation of multiplying the output frequency by the current gain. However, the voltage output of the RO does not fully swing, since the bottom n-type metal-oxide-semiconductor (nMOS) source transistors (M_10_ and its correspondents) are connected to the nMOS of the biasing circuit (M_6_) instead of directly to the ground level. To produce the rail-to-rail output by applying the skewed buffer to the RO output in a 180 nm process with a 1.8 V power source, the output voltage level of the RO should swing from 1.8 to 0.8 V for the proper operation of the circuit considering the threshold variation. To achieve this outcome, the resistance of M_6_ should be set relatively lower than that of the overall RO.

**Figure 4:**
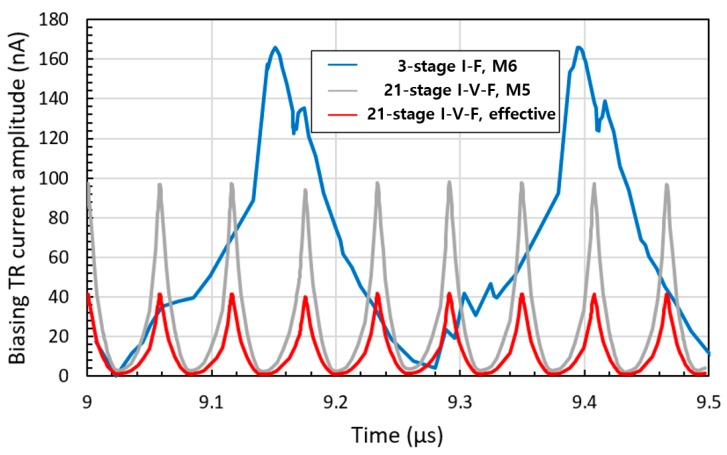
Current fluctuation of the biasing transistors.

One of the main causes of linearity error is current fluctuation in the biasing transistor M_6_. As RO oscillates, each stage of the RO is in a different operating state since the gate voltages differ. The overall current of the RO is the sum of the current of each stage. As the output voltage of the stacked inverter is not directly proportional to the gate voltage, the sum of the current flowing through each stage is not fixed in the oscillating cycle. The current in the biasing transistor (TR) should be consistent, as fluctuation in the biasing TR directly affects the frequency of the RO. However, as the biasing circuit is connected to the RO, the fluctuation in the overall RO current induces fluctuation in the biasing TR, as shown in Figure 4.

**Figure 5:**
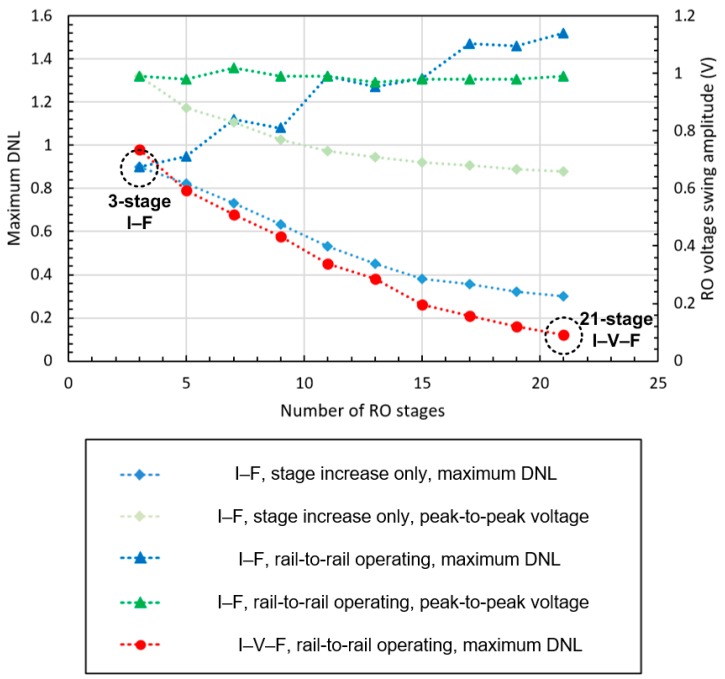
Maximum differential nonlinearity (DNL) in 1 nA steps, and RO voltage swing amplitude according to the number of the RO stages.

Increasing the number of RO stages reduces the overall RO current fluctuation. As the current difference between stages decreases, the current fluctuation in the biasing transistor decreases. The decrease in DNL caused by increasing the number of RO stages is shown in Figure 5. However, in this circuit configuration, as the number of the stages increases, the RO peak-to-peak voltage decreases since the operating resistance of the RO decreases while M6 resistance remains constant.

To compensate for the peak-to-peak voltage loss, the width of the M_6_ needs to be increased. As the resistance of M_6_ decreases, M_6_ becomes less tolerant to changes in RO current flow. As shown in Figure 5, as the number of RO stages increases, the linearity error increases while maintaining the rail-to-rail operation. Thus, the three-stage I–F structure was selected for comparison with the proposed I–V–F converting current transducer.

## 3. Proposed I–V–F Converting Current Transducer

**Figure 6:**
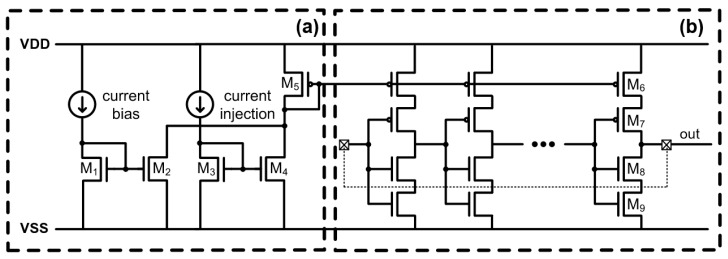
Proposed I–F converter with (**a**) cascade bias circuit and (**b**) RO with biased delay cells.

To overcome the drawbacks of the previously developed RO-based current transducer, a novel RO-based I–V–F converter, achieved using the simple CMOS logic architecture, is proposed for measuring current, having the advantages of compact area and low power consumption. Figure 6 shows the proposed converter architecture based on an I–V–F converting ring oscillator. The transducer is composed of a stacked inverter (M_6_–M_9_)-based RO, with a top p-type metal-oxide-semiconductor (pMOS) (M_6_ and correspondents) gate biased by a cascade biasing circuit.

The cascade biasing circuit is composed of the initial biasing circuit (M_1_, M_2_ and the self-bias structure as in an I–F converting transducer) and the biasing circuit that operates according to the current injection (M_3_, M_4_). M_1_ and M_2_ (M_3_ and M_4_) compose the typical current mirror, and M_5_ was added for second-stage mirroring. M_5_ converts the mirrored current data (the sum of current flowing in M_2_ and M_4_) into the voltage data for RO biasing. As the injected current increases, the voltage level of the pMOS (M_6_ and correspondents) gates in the delay cells decrease, causing the oscillation frequency to increase proportionally to the injected current, as shown in Figure 3b.

As the current is not directly injected to the RO, different from the I–F converting current transducer, the biasing pMOS TRs can be assigned to each stage. The bottleneck of the large biasing TR for rail-to-rail operation is thus removed. Therefore, increasing the number of RO stages does not decrease the RO peak-to-peak voltage and improves the linearity characteristic while maintaining the rail-to-rail operation, as shown in Figure 5. The number of the RO stages was set to 21.

As each delay cell is controlled by the gate voltage level of M_6_ set by the pMOS (M_5_) of the cascade bias circuit, the biasing TR (M_5_ in Figure 6) is less affected by the RO than that of the I–F converting current transducer (M_6_ in Figure 2) since the drain of the biasing TR in the I–F converter is connected to the source of the RO. The current fluctuation of the biasing TRs of the two methods are shown in Figure 4. Compared with the I–F scheme, where the amplitude of current fluctuation in the biasing TR is equal to that of the injected current, a 1 nA increase in the injected current causes a 2.35 nA increase in the biasing current. Thus, the effective amplitude of current fluctuation is 2.35 times smaller than that of the biasing TR current fluctuation, as shown in Figure 4 (I–V–F, effective).

## 4. Results and Discussion

**Figure 7:**
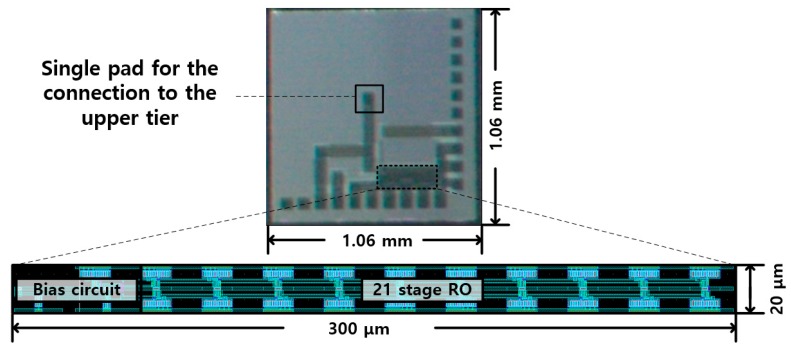
Photograph of the fabricated chip.

**Figure 8:**
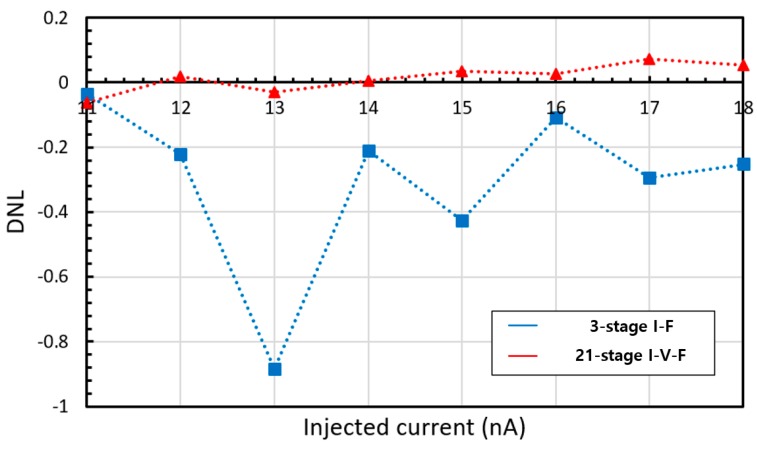
DNL in 1 nA steps of the I–V–F converting current transducer.

**Figure 9:**
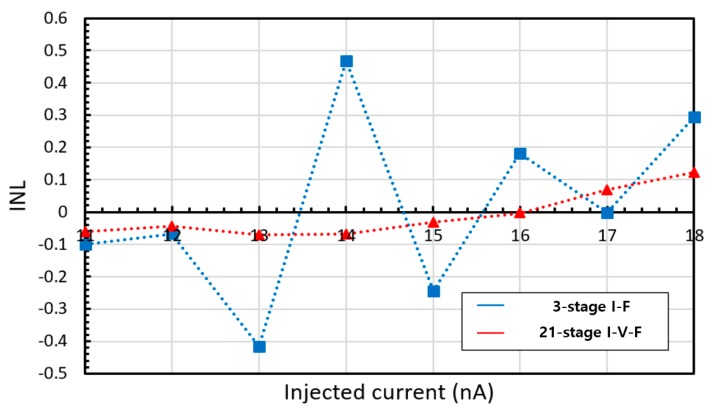
Integral nonlinearity (INL) in 1 nA steps of the I–V–F converting current transducer.

**Figure 10:**
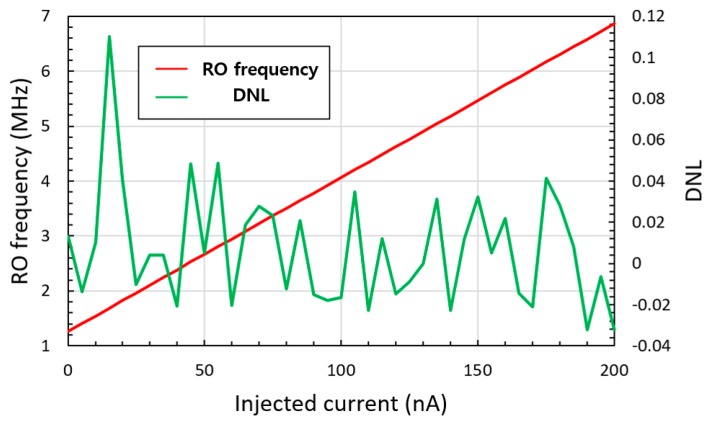
Frequency output and DNL in 1 nA steps of the 21-stage I–V–F converting current transducer over the 200 nA input current range, with 5 nA plot step.

An RO-based I–F converting current transducer was fabricated using NNFC 180 nm CMOS technology, as shown in Figure 7, where a 21-stage RO and cascade bias circuit were used. The overall chip size is 1.06 × 1.06 mm with an effective area of 300 × 20 μm. The current transducer can be integrated into biosensors in three dimensions (3D), with a current input path of 60 × 60 μm to the upper current-emitting device. From Figure 4, the maximum amplitude of current fluctuation in the biasing TR of I–V–F is 41.8 nA, which is 3.93 times lower than that in the I–F, which is 164.2 nA. As mentioned above, the lower current fluctuation amplitude originates from the higher number of RO stages and the higher output resistance of the bias circuit. The difference in the current fluctuation results in different output characteristics of the current sweep, as shown in Figure 3. The output frequency of the proposed transducer exhibited better linearity, as shown in Figure 8 and Figure 9. The maximum DNL measured at a current step of 1 nA is <0.15 nA over the input current range of 0 to 20 nA, which is nine times less than the DNL of I–F. Figure 10 shows the RO frequency output of the proposed structure, with DNL in 1 nA steps over a 200 nA range. This result yields an output gain of 27.3 kHz/nA and a current resolution of 1 nA over the full-scale range of 200 nA. The rms value of the output frequency is 0.015 kHz. From this rms value and the output gain determined above, the noise floor was calculated to be 11.8 pA/√Hz. As the proposed transducer is based on RO, the frequency gain of the transducer changes according to the process, voltage, and temperature (PVT) variation, as in Table 1. However, as the simple RO structure ensures the sublinear sweep of the output frequency, the frequency gain is calculable with the measured output frequency at 0 nA input, which is derived and simplified from the simulation result of the PVT analysis.

$$Frequency\;gain\;\left(MHz/nA\right)=\frac{output\;frequency\;at\;0\;nA\;input\;\left(MHz\right)}{45.68\;nA}$$

**Figure 11:**
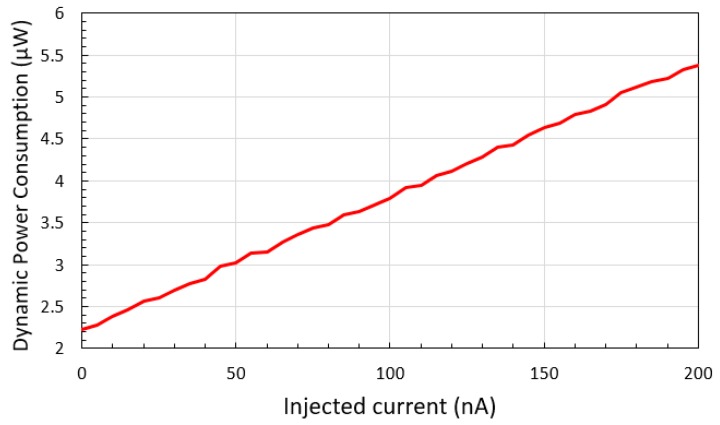
Dynamic power consumption of the I–V–F converting current transducer.

The maximum DNL in 1 nA steps remains under 0.2 nA in any PVT variation condition given, which means that the output linearity characteristic of the proposed converter is tolerant against the PVT variation. The transducer is activated with a 1.8 V power supply, and the dynamic power consumption is 2.16 μW at 0 nA input, up to 5.44 μW at 200 nA input, as shown in Figure 11. The transducer performance measurement was done by the ideal current source, which has a high output resistance. The resistance of the TR M_3_ in Figure 3 varies up to 1 Mohm at 200 nA current injection. For the integration of the current-emitting device with the proposed I–V–F converting current transducer, the output resistance of the device should be over 600 Mohm to maintain the 1 nA output resolution.

**Figure 12:**
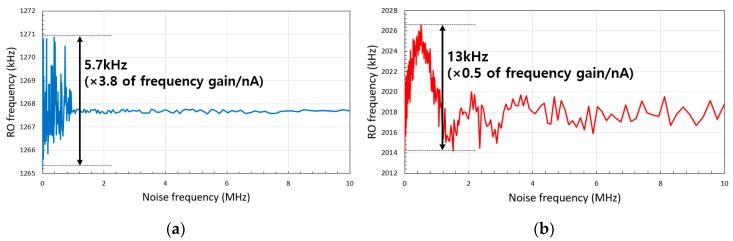
Frequency output of the (**a**) three-stage I–F and (**b**) 21-stage I–V–F converting current transducer according to the noise frequency at 30 nA current injection and 1 nA noise amplitude.

When the converting current transducer is integrated with the current-emitting device, the intrinsic noise from the device will affect the RO biasing voltage. The sinusoidal noise simulation result of two types of transducers at 30 nA current injection and 1 nA noise amplitude is shown in Figure 12. The maximum difference of the RO frequency is about 5.8 kHz and 13 kHz, which correspond to 3.8 nA and 0.5 nA injected current in I–F and I–V–F converting current transducer, respectively. The I–V–F converting current transducer reduces the input-referred noise amplitude by 50% or more as the RO frequency is an averaged output. Although the I–F converting current transducer also uses the RO for transducing, the effect of the noise to the output frequency sub-linearly depends on the DNL. Thus, the 1 nA noise of the I–F converting current transducer affects the output frequency more than that of the I–V–F converting current transducer, as the maximum DNL of the I–F converting current transducer is six times higher than that of the I–V–F converting current transducer, as shown in Figure 5.

## 5. Conclusions

In this paper, a simple but well-functioning I–V–F converting current transducer is introduced. Area compactness and low power consumption are the two main criteria in the supplementary circuit for the bio-sensing applications. The proposed circuit was designed to minimize the area and power while ensuring sufficient linearity characteristics of measurement by modifying the conventional RO based converting scheme. This compact area, rail-to-rail output, low noise floor transducer with monolithic-3D integration applicability has significant potential for improving bio-sensing technologies.

## Figures and Tables

**Table 1:** Simulated frequency gain and maximum DNL in 1 nA steps according to the PVT variation.

**Process Corner**	**SS**	**SF**	**TT**	**FS**	**FF**
Frequency gain (kHz/nA)	26.701	27.275	27.299	27.356	27.653
Maximum DNL	0.1237	0.1123	0.1145	0.1186	0.1423
**Temperature**	**0 °C**	**25 °C**	**50 °C**	**75 °C**	**100 °C**
Frequency gain (kHz/nA)	27.267	27.299	27.334	27.384	27.447
Maximum DNL	0.1167	0.1145	0.1153	0.1186	0.183
**Supply Voltage**	**1.62 V**		**1.8 V**		**1.98 V**
Frequency gain (kHz/nA)	27.132		27.299		27.532
Maximum DNL	0.1327		0.1145		0.1246
